# Association between Adverse Childhood Experiences and Diagnosis of Cancer

**DOI:** 10.1371/journal.pone.0065524

**Published:** 2013-06-11

**Authors:** Monique J. Brown, Leroy R. Thacker, Steven A. Cohen

**Affiliations:** 1 Department of Family Medicine and Population Health, Virginia Commonwealth University School of Medicine, Richmond, Virginia, United States of America; 2 Department of Biostatistics, Virginia Commonwealth University School of Medicine, Richmond, Virginia, United States of America; 3 Center for Clinical and Translational Research, Virginia Commonwealth University School of Medicine, Richmond, Virginia, United States of America; University College London, United Kingdom

## Abstract

**Objective:**

Adverse childhood experiences (ACEs) are linked to multiple adverse health outcomes. This study examined the association between ACEs and cancer diagnosis.

**Methods:**

Data from the 2010 Behavioral Risk Factor Surveillance System (BRFSS) survey were used. The BRFSS is the largest ongoing telephone health survey, conducted in all US states, the District of Columbia, Puerto Rico, Guam and the U.S. Virgin Islands, and provides data on a variety of health issues among the non-institutionalized adult population. Principal component analysis (PCA) was used to derive components for ACEs. Multivariable logistic regression models were used to provide adjusted odds ratios (OR) and 95% confidence intervals (CI) for the association between ACE components and overall, childhood and adulthood cancer, adjusting for confounders such as age, gender, race/ethnicity, income, educational status, marital status, and insurance status.

**Results:**

Approximately 62% of respondents reported being exposed to ACEs and about one in ten respondents reported ever having been diagnosed with cancer. Component 1, which had the sexual abuse variables with the highest weights, was significantly associated with adulthood cancer (adjusted OR: 1.21; 95% CI: 1.03–1.43).

**Conclusion:**

The association between ACEs and adulthood cancer may be attributable to disease progression through association of ACEs with risk factors for other chronic diseases. More research should focus on the impact of sexual abuse ACEs and adverse health outcomes.

## Introduction

Adverse childhood experiences (ACEs) represent a child’s exposure to negative events, including emotional, physical and sexual abuse, domestic violence, absence of a parent because of divorce or separation, and a family/household member’s mental illness, incarceration, and/or substance abuse [1], [2]. Research has shown that majority of adults have experienced at least one ACE, [3] with prevalence estimates ranging from 65% to 87% [4], [5]. The Centers for Disease Control and Prevention (CDC) has found that 59.4% of Behavioral Risk Factor Surveillance System (BRFSS) respondents in 2009 reported having at least one ACE, approximately 25% of adults reported experiencing ≥3 ACEs and 8.7% reported ≥5 ACEs [2]. These high prevalence estimates highlight the importance for additional efforts, locally, state-wide, and nationally, to help in the reduction and prevention of child maltreatment, and associated family dysfunction. There is also a need for services to treat outcomes, especially stress-related health illnesses, associated with ACEs [2], from childhood through adulthood.

Research has shown that ACEs are linked to multiple adverse health outcomes, and are interrelated rather than occurring independently [6]. ACEs have been linked to substance abuse [7]–[11], depression [7],[8],[12],[13], cardiovascular disease [7],[14], diabetes [7], cancer [7],[15],[16], risky sexual behaviors [Bibr pone.0065524-Brown2] [Bibr pone.0065524-Klein1] [17–19], sexually transmitted infections [7],[8],[19] suicidality [7],[8],[13],[17] [18], and premature mortality in adulthood [17]. Researchers have suggested examining multiple ACEs allowing for a potential assessment of a graded relationship between these exposures and health outcomes [6]. However, exploring ACEs using principal component analysis (PCA), a more robust method, will account for the loading of each adverse experience. Using this strategy will allow for aggregation of ACE components that are likely to cluster together.

Stressful and/or traumatic experiences during childhood may also have neurodevelopmental impacts that persist over the lifespan [7]. The public health impact of ACEs has only been recently evaluated systematically through large epidemiologic efforts [20]. A strong relationship has been shown between stress and/or trauma during childhood, and smoking behavior [21],[22] with a threefold increase in the risk of lung cancer for participants who had ≥6 ACEs [15]. However, the increase in the risk of lung cancer could only be partially explained by the relationship between ACEs and smoking. Therefore, this finding suggests that the association between ACEs and cancer may be attributable to other mechanisms in which stressors and trauma during childhood negatively affect health [15]. In a prospective study, adverse events occurring between ages six and eight, and cumulative adversity from birth to age eight were found to be associated with inflammation at age ten [23].

While the link between ACEs and adverse health outcomes, including lung cancer, has been demonstrated, the association between ACEs and diagnosis of all cancers has not been examined. The main aims of this study were to: 1) Examine the association between ACEs and all cancers; and 2) Examine the association between ACEs, and childhood cancer and adulthood cancer. We hypothesize that we will see a positive association between ACEs, and overall, childhood and adulthood cancer. To our knowledge, this is the first study to examine the association between ACEs and all cancers, childhood and adulthood cancer using a population-based sample.

## Methods

### Ethics Statement

The Virginia Commonwealth University Institutional Review Board provided a formal written waiver for the current study as publicly available, anonymous, secondary data were used. Verbal consent was provided to interviewers for the Behavioral Risk Factor Surveillance System survey [25].

### Data Source and Sample

This cross-sectional study used data from the 2010 Behavioral Risk Factor Surveillance System (BRFSS) survey. The BRFSS is the largest ongoing telephone health survey, conducted in all US states, the District of Columbia, Puerto Rico, Guam and the U.S. Virgin Islands [24]. Established by the CDC, the BRFSS provides data on a variety of health issues among the non-institutionalized adult population (age 18 years and older). In addition to core questions that are asked by every state, there are optional modules that cover other health topics or ask more in-depth questions on a topic that was included among the core questions. The 2010 survey included an optional ACE module [25]. Wisconsin was the only state that also included the cancer survivorship module [25]. Respondents in Wisconsin who refused, or were not asked questions relating to ACEs and cancer diagnosis were not eligible (n = 551). The resultant sample size was 4,230.

### Operational Definition of Adverse Childhood Experiences

The optional ACE module included questions about adverse, stressful and/or traumatic events experienced as a child (Table 1). We created three component scores of the ACEs using principal component analysis (PCA). The use of adverse events during childhood in research has shown to be dependable as the test-retest reliability in the responses to questions about ACEs has been found to be good to excellent [26].

**Table 1:** Types of Adverse Childhood Experiences, related BRFSS questions and operational definitions.

Questions	Operational Definition	Abbreviation
1) Did you live with anyone who was depressed, mentallyill or suicidal?	No vs. Yes	DEPRS
2) Did you live with anyone who was a problem drinkeror alcoholic?	No vs. Yes	DRINK
3) Did you live with anyone who used illegal street drugsor who abused prescription medications?	No vs. Yes	DRUGS
4) Did you live with anyone who served time or wassentenced to serve time in a prison, jail, or othercorrectional facility?	No vs. Yes	PRISN
5) Were your parents separated or divorced?	No vs. Yes	DIVRC
6) How often did your parents or adults in your homeever slap, hit, kick, punch or beat each other up?	Never vs. At least once	PUNCH
7) How often did a parent or adult in your home ever hit,beat, kick, or physically hurt you in any way? Do notinclude spanking.	Never vs. At least once	HURT
8) How often did a parent or adult in your ho0me everswear at you, insult you, or put you down?	Never vs. At least once	SWEAR
9) How often did anyone at least 5 years older than you oran adult, ever touch you sexually?	Never vs. At least once	TOUCH
10) How often did anyone at least 5 years older than you oran adult, try to make you touch them sexually?	Never vs. At least once	TTHEM
11) How often did anyone at least 5 years older than youor an adult, force you to have sex?	Never vs. At least once	HVSEX

### Operational Definition of Cancer

The Cancer Survivorship module, which was also optional, included several questions on cancer diagnosis and treatment. Cancer was selected as previous research has suggested a link between ACEs and different types of cancer [Bibr pone.0065524-Brown1] [15,16]. Cancer diagnosis was defined by: 1) “Have you ever been told by a doctor, nurse, or other health professional that you had cancer?” which elicited a binary (yes/no) response; and 2) “At what age were you told that you had cancer?” Age of cancer diagnosis was then separated into 1–17 years (childhood cancer) [Bibr pone.0065524-Gupta1] [Bibr pone.0065524-Stavrou1] [27–29] and 18 years or older (adulthood cancer). Self-reported data on cancer diagnosis has been shown to have high validity with moderate to excellent agreement to medical chart information, and has been shown to be useful for epidemiologic research [Bibr pone.0065524-Gupta1] [27,28].

### Potential Confounders

Potential confounders considered were either associated with ACEs or cancer diagnosis, as reported in the literature. In previous studies, sociodemographic variables were included as covariates [20]. Older age is a risk factor for cancer [Bibr pone.0065524-Centers4] [29,30] and age was included as a continuous covariate. Sex and race/ethnicity were also included as covariates in the models as they are strong predictors for certain types of cancers. For example, aside from non-melanoma skin cancer, breast cancer is the most common cancer among women, and prostate cancer is the most common cancer among men [Bibr pone.0065524-Centers6] [Bibr pone.0065524-Centers7] [31–33]. From 1999 to 2008, White women had the highest breast cancer incidence rates while Black women had the highest mortality rates [34]. Men tend to have higher colorectal cancer incidence and mortality rates compared to women with Black men having higher colorectal cancer incidence and mortality rates compared to men of other racial/ethnic groups [35]. Income, educational level, and marital status have also been included in previous studies as a covariate examining the link between ACEs and adverse outcomes [Bibr pone.0065524-Brown1] [15,20]. Socioeconomic status may partially explain the association of traumatic events during childhood with chronic illness [36]. Insurance status was also considered as a potential confounder as adverse experiences during childhood is linked to health [37], which is ultimately influenced by access to healthcare and insurance status.

### Analytic Approach

All analyses considered the BRFSS complex multistage sampling strategy [38]. Weighted prevalence estimates were obtained for ACEs and cancer. Proc surveyfreq procedures were used for bivariate analyses to examine the association between age, gender, race/ethnicity, income, educational status, marital status and insurance status, and exposure to ACEs (Table 2). P values were used to determine statistically significant differences between exposure to overall ACEs and no exposure to ACEs.

**Table 2:** Distribution of socio-demographic characteristics of BRFSS respondents by Exposure to Adverse Childhood Experiences.

Confounders	ACEs	No ACEs	P-value*
	N = 2,505	N = 1,725	
	WN = 2,463,391	WN = 1,490,356	
	%	%	
**Age**			
18–34	36.6	20.1	<0.0001
35–49	25.8	24.8	
50+	37.6	55.1	
**Gender**			
Female	50.5	50.6	0.9689
Male	49.5	49.4	
**Race/Ethnicity**			
White	87.6	93.5	0.0001
Black	3.5	1.6	
Hispanic	3.0	0.8	
Other^a^	5.9	4.2	
**Income (Annual)**			
<$15,000	5.4	2.1	<0.0001
$15,000–<$50,000	52.2	44.8	
$50,000+	42.4	53.0	
**Education** b			
<HS Graduate	5.0	4.6	0.1437
HS Graduate	32.9	29.2	
>HS Graduate	62.1	66.2	
**Marital Status**			
Married	58.8	69.5	<0.0001
Not married	41.2	30.5	
**Insurance Status**			
Insured	87.1	93.8	<0.0001
Not insured	12.9	6.2	

N = Frequency; WN = Weighted frequency.

* P-value shows differences between respondents exposed to ACEs and unexposed to ACEs.

a The group “Other” contains respondents who identified themselves as “Multiracial”, “Other”, “Native American/Alaska Native” and “Native Hawaiian/Other Pacific Islander”.

b Education categories: <High school graduate, High school graduate, >High school graduate.

PCA was used to obtain a score (Component 1) taking into consideration the shared information across the ACEs; and shape components (Components 2 and 3) that were independent of Component 1. We observed the component loading pattern (the correlation coefficients of the original variables with the components) (Table 3). Multivariable logistic regression models were used to provide adjusted odds ratios (OR) and 95% confidence intervals (CI) for the association between the three principal components of ACEs and overall cancer, childhood cancer, and adulthood cancer adjusting for age, gender, race/ethnicity, income, educational status, marital status, and insurance status (Table 4). A variable was considered to be a potential confounder based on the literature review done *a priori,* as well as analyses shown in Table 2 comparing individuals exposed to ACEs to those not exposed to ACEs.

**Table 3:** Adverse Childhood Experiences Pattern derived by Principal Component Analysis.

Component 1		Component 2		Component 3	
ACE	Load	ACE	Load	ACE	Load
TOUCH	0.40	DRUGS	0.31	SWEAR	0.52
TTHEM	0.40	DRNK	0.31	HURT	0.46
HVSEX	0.38	PUNCH	0.29	DIVRC	0.37
HURT	0.30	PRISN	0.28	TOUCH	0.03
PUNCH	0.30	DIVRC	0.25	DRUGS	−0.46
DRUGS	0.29	DEPRS	0.20	PRISN	−0.41
DRNK	0.28	HURT	0.14	PUNCH	−0.06
DEPRS	0.26	SWEAR	0.07	HURT	−0.05
DIVRC	0.25	TOUCH	−0.44	SWEAR	−0.04
PRISN	0.24	TTHEM	−0.42	TTHEM	−0.02
SWEAR	0.07	HVSEX	−0.39	HVSEX	−0.01

**Table 4:** Multiple Logistic Regression of Principal Components of Adverse Childhood Experiences with Cancer Diagnosis in Adulthood.

Component	Overall		Childhood		Adulthood	
	cOR^a^	aOR^b^	cOR^a^	aOR^b^	cOR^a^	aOR^b^
	(95% CI)	(95% CI)	(95% CI)	(95% CI)	(95% CI)	(95% CI)
**Component 1**	1.02	1.15	1.35	0.75	1.02	**1.21**
	(0.88–1.17)	(0.97–1.37)	(0.47–3.91)	(0.48–1.18)	(0.89–1.18)	**(1.03–1.43)**
**Component 2**	0.90	1.06	1.05	0.88	0.86	1.03
	(0.77–1.06)	(0.86–1.31)	(0.76–1.45)	(0.43–1.81)	(0.74–1.01)	(0.83–1.29)
**Component 3**	1.10	0.95	1.35	2.52	1.10	0.93
	(0.88–1.39)	(0.76–1.18)	(0.47–3.91)	(0.47–13.4)	(0.87–1.39)	(0.74–1.17)

a Crude odds ratio.

b Adjusted odds ratio controlling for age, gender, race/ethnicity, income, educational status, marital status, and insurance status.

## Results

### Weighted Prevalence Estimates

The weighted prevalence of ACEs was 62.3%. Approximately 10% of respondents reported having had a diagnosis of cancer, with 9.3% reporting their first diagnosis of cancer in adulthood and 0.2% reporting their first diagnosis of cancer during childhood. Among respondents who reported a diagnosis of cancer, 98.5% reported having their first diagnosis during adulthood (at age 18 or older).

### Distribution of Characteristics across Exposure Groups

Table 2 shows the distribution of socio-demographic characteristics of BRFSS respondents by exposure and non-exposure to ACEs. There were statistically significant differences in the proportions of respondents who reported ACEs compared to those who did not by age, race/ethnicity, income, education, marital status, and insurance. More than half of the respondents who did not report exposure to ACEs were 50 years or older. Of the respondents who reported ACEs, 87.6% were White compared to 93.5% of the respondents who did not report ACEs. Among those respondents who reported ACEs, a higher percentage of Black, Hispanic or Other respondents reported ACEs compared to respondents in these racial/ethnic groups who did not report ACEs. There was no statistically significant difference by gender. Approximately a third of respondents who reported ACEs were high school graduates and just under a third of respondents had at least some college education.

### Component Loading Pattern

Table 3 shows the ACE pattern derived from PCA. All the values for each ACE were positively correlated with Component 1. The sexual abuse variables (TOUCH, TTHEM and HVSEX) had the highest weights for Component 1. Variables that indicated ACEs that were not directed towards the child (DEPRS, DRINK, DRUGS, PRISN, DIVRC, and PUNCH) had the highest positive weights for Component 2; and the two variables that indicated psychological and physical abuse towards the child (HURT and SWEAR, respectively) had the highest positive weights for Component 3.

### Association between ACEs and First Diagnosis of Cancer

Table 4 shows the association between ACEs and diagnosis of all cancers, childhood cancer and adulthood cancer. There were no statistically significant results using the unadjusted models. However, after adjusting for age, sex, race/ethnicity, income, education and marital status, Component 1 was significantly associated with being diagnosed with cancer in adulthood (adjusted OR: 1.21; 95% CI: 1.03–1.43). Components 2 and 3 were not significantly associated with diagnosis of cancer.

## Discussion

While many studies have reported on ACEs and specific types of cancer, to our knowledge, no study has reported on the association between ACEs and all cancers, differentiating between cancer in childhood and adulthood. The complex sampling scheme also provides population-based estimates. Among the respondents, approximately one in ten respondents reported ever having been diagnosed with cancer. The prevalence estimate of cancer derived from the current study of 10% is higher than the estimate of 4.2% derived from the National Cancer Institute (NCI) [Bibr pone.0065524-American1] [39,40] and census population estimates [41]. Component 1, for which the sexual abuse variables had the highest weights, was significantly associated with cancer in adulthood.

This is the first study to examine adverse childhood experiences using PCA. By using PCA, we were able to use the data to derive three components of ACEs. Brown et al. (2010) found a positive association between ACEs and risk of lung cancer, which coincides with the findings in the current study. However, the current study considered prevalence of all cancers overall, in childhood and adulthood, in a cross-sectional study with a population-based sample while Brown et al. (2010) looked at incident lung cancer using a prospective study design [15].

Evidence suggests that ACEs have been associated with risky sexual behaviors, [17]–[19] sexually transmitted infections, [7] [8],[19] chronic diseases such as cancer, [15],[16] obesity, [42] depression [7],[8],[12],[13], and smoking [42],[43]. Research has also shown that ACEs are associated with risk factors for chronic disease [15] such as chronic obstructive pulmonary disease [44], liver disease [4], and ischemic heart disease [14]. ACEs can be viewed as an initial exposure that may result in adverse health outcomes during adulthood. However, exposure to adversity during childhood is a likely source of, not only chronic but also acute stressors. [45] These acute stressors may alter fundamental biological functions, therefore, adversely impacting health, even before the onset of adulthood. In utero exposure to adverse conditions can result in adverse health outcomes [46] throughout the life course. However, in the current study, we did not find an association between ACEs and childhood cancer. These findings suggest that ACEs may be related to adulthood cancer via alternate pathways as at present, we do not have biological models linking ACEs to cancer. One possible pathway is through the association of ACEs with risk factors for chronic diseases that may negatively affect general health and contribute to disease progression [15], which can predispose an individual for cancer in adulthood. However, more research is needed to explore this potential mechanism.

This study must be considered with limitations in mind. First, the study was cross-sectional. Based on the survey questions, we were not able to determine age of exposure to adverse childhood experiences. Therefore, it is possible that a diagnosis of cancer in childhood could have occurred before exposure to ACEs. However, since the majority of cancers were adulthood cancers (98.5%), the ambiguity of temporal sequence would have only applied to 1.5% of the cancer cases in the overall category. It is also possible that there could have been underreporting of ACEs due to desirability bias, which would result in non-differential misclassification of the exposure. However, if this were the case, then the estimates produced would have been underestimates of the “true” association.

The study also had several strengths. We are able to obtain population-based estimates of the prevalence of cancer and exposure to ACEs using a comprehensive, nationally representative database. This is the first study to use PCA to assess the association of ACEs with an adverse health outcome, and the first study to examine ACEs and overall, childhood and adulthood cancer. The use of PCA also allowed for aggregation of variables that were likely to cluster together without relying on subjective construction of factors.

### Conclusions

The results show an association between Component 1, for which the sexual abuse had the highest weights, and adulthood cancer. A reduction in ACEs, perhaps sexual abuse ACEs, could reduce adverse health outcomes, such as cancer. ACEs screening should be implemented during routine healthcare examinations for children and adults so as to help in identifying patients who may be at risk for chronic illnesses. Future research should focus on sexual abuse ACEs and other adverse health outcomes to determine if individuals who experienced these specific types of ACEs would be at risk for other illnesses.
