# Correction: Association between Adverse Childhood Experiences and Diagnosis of Cancer

**DOI:** 10.1371/annotation/bd99c401-8d86-465a-a930-bd84bb662657

**Published:** 2014-01-29

**Authors:** Monique J. Brown, Leroy R. Thacker, Steven A. Cohen

There is an error in the last two sentences of the sub-heading "Data Source and Sample" under the "Methods" section. The correct sentences for this section are:

"Respondents in Wisconsin who refused, or were not asked questions related to ACEs were not eligible (618). The resultant sample size was 4,163."

Additionally, there is an error in the first sentence of the sub-heading "Weighted Prevalence Estimates" under the "Results" section. The correct sentence for this section is:

"The weighted prevalence of ACEs was 63.1%."

Finally, there is an error in Table 2. Please view the correct Table 2 here: http://www.plosone.org/corrections/pone.0065524.t002.cn.docx

